# Silk-Derived Peptide Modification to Polymers Improves the Miscibility of Composite Materials with Silk Fibroin

**DOI:** 10.1021/acsomega.5c05282

**Published:** 2025-10-24

**Authors:** Yuri Matsumoto, Shota Akioka, Yasumoto Nakazawa

**Affiliations:** Department of Biotechnology and Life Science, Tokyo University of Agriculture and Technology, 2-24-16 Naka-cho, Tokyo 184-8588, Japan

## Abstract

Silk fibroin (SF)/polymer composite materials typically exhibit degraded material strength because of their low miscibility. Therefore, we hypothesize that compatibility issues can be solved by modifying SF primary structure motif peptides into polymers. Physical property and structural analyses are performed on fabricated SF/polymer composites. These results suggest that miscibility is improved by modifying the peptide in terms of its thermal properties and molecular mobility. Further, physical properties are improved by peptide modification and are correlated with molecular structure. These findings indicate that SF primary structure motif peptide modification is an innovative technology that can solve the miscibility issue of SF/polymer composite materials.

## Graphical abstract

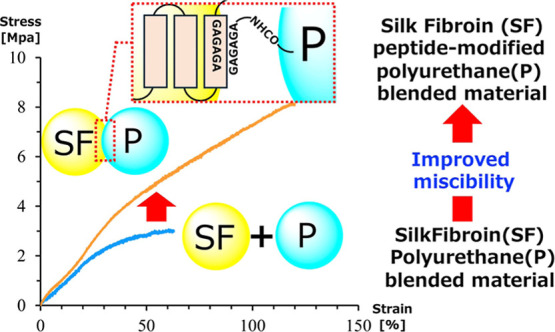

## 1 Introduction

Silk is a natural fiber protein produced by silkworms, spiders, and bagworms, and its mechanical properties and functionality vary depending on the type.^1–3^ Silk from domesticated silkworms (*Bombyx mori*) has been widely studied owing to its superior tensile properties, modulus, toughness, and elongation in addition to its high production quantity and availability.^4,5^ Silk includes two types of fibrous proteins, and the inner two fibrous proteins are called silk fibroin (SF).^4,5^ SF has very high mechanical strength because it forms a β-sheet structure,^4^ and it is highly suitable as a biomaterial because of its mild biodegradability,^6^ low inflammatory, and blood and tissue compatibility. Furthermore, its easy structural reformation and high formability^7^ enables SF to be a multipurpose material for tissue engineering,^6^ food engineering,^8^ plastic engineering,^9^ and other applications. However, compared to natural silk fibers, regenerated SF materials formed by dissolving and molding SF have less optimized secondary structures, thereby resulting in degraded mechanical properties.^6^ Therefore, many studies have focused on reinforcing the mechanical properties by blending with other polymers.

Polymer blending is the simplest way to modify material properties, and polymer blends have been used for a long time. However, in many cases, the theoretically predicted modification effect of material properties has not been observed.^10^ The high interfacial tension between separated phases is caused by the large size of polymer molecular chains or the lack of adhesion between polymer phases, resulting in immiscibility.^10–12^ Clear phase separation conditions have been observed in polymer blend materials that exhibit low miscibility,^13^ which degrades physical properties and affects degradability.^14,15^ Natural proteins such as SF have higher surface energy and aggregate more easily than synthetic polymers, reducing their miscibility, especially with nonpolar polymers.^16^ Currently, to the best of our knowledge, there are often examples of studies analyzing the miscibility between SF and certain polymers (e.g., poly lactic acid,^17^ sodium alginate,^18^ chitosan,^19^ etc.), but research on universally improving miscibility with SF regardless of polymer species is clearly lacking.

In this study, we focused on the characteristic primary structure of SF to develop a new method that can improve the miscibility of SF/polymer composite materials. SF is composed of amino acids such as glycine (Gly, G), alanine (Ala, A), and serine (Ser, S), and its structure can be roughly divided into the crystalline region (∼80%) and the amorphous region (∼20%).^1,7^ Crystal regions are dispersed throughout the SF primary structure, with ∼94% of them taking a G-X repeat sequence: X indicates amino acids, containing 64% A, 22% S, and ∼10% tyrosine (Tyr, Y). This repetitive sequence is referred to as (GX)_*n*_.^1^ It forms a strong crystal structure based on a secondary structure, mainly the β-sheet structure, via intra/inter-SF molecular hydrogen bonds, hydrophobic interactions, and van der Waals forces.^7^ Based on the above structural features, we hypothesize that the miscibility issue can be solved by modifying (GX)_*n*_ motif peptides to the polymer that mixes with SF. The improved interfacial adhesion caused by packing between the SF molecular chain and polymer-modified (GX)_*n*_ motif peptides would suppress phase separation, improving miscibility. So, two (GX)_*n*_ motif peptides, GAGAGA (AAA) and GAGYGA (AYA), have been used in this study.

Water-dispersed polyurethane (WPU) was used as a polymer to be mixed with SF, which is a polymer with excellent strength and flexibility. Moreover, WPU has good water dispersibility because of the abundant carboxyl groups, which are highly reactive functional groups, in the PU structure and the incorporation of hydrophilic polymers. This dramatically increased the efficiency of peptide modification, which is why it was used in this study.

In this paper, we discuss the improvement of miscibility by peptide modification to polymer based on structural and physical properties analysis.

## 2 Materials and Methods

### 2.1 Preparation of a Silk Fibroin Aqueous Solution

Silk fibers were obtained from *Bombyx mori* cocoons and degummed with respect to previous report.^20^ Raw silk was reeled and woven silk cocoons were boiled in hot water at 80 °C for ∼15 min. The dried silk threads were immersed in a 0.02 M sodium carbonate (Na_2_CO_3_, Wako Pure Chemical Industries Ltd., Tokyo, Japan) aqueous solution and stirred at 90 °C for 30 min. After silk sericin was removed by rinsing it many times with ultrapure water at 40 °C, pure SF was obtained by standing at 37 °C for 24 h. The SF fibers were dissolved in 9 M lithium bromide (LiBr, Wako Pure Chemical Industries, Ltd., Tokyo, Japan) solution at a concentration of 10% (w/v) at 37 °C for 1 h. Then, this solution was dialyzed against ultrapure water, until LiBr was completely removed. Contaminants were removed by centrifugation of the dialyzed solution to purify the SF aqueous solution. The final concentration of the aqueous SF solution was ∼4% (w/v).

### 2.2 Peptide Modification to Polymer

Peptides were cross-linked to WPU (Tosoh Co., Tokyo, Japan) using *N*-(3-(dimethylamino)­propyl)-*N*′-ethylcarbodiimide hydrochloride (EDC, Thermo Fisher Scientific K.K., Tokyo, Japan) and sulfo *N*-hydroxysuccinimide (sNHS, Thermo Fisher Scientific K.K., Tokyo, Japan). After the WPU water dispersant was diluted to 1% (w/v) using a 0.05 M buffer of 2-morpholinoethanesulfonic acid (MES, Dojindo Laboratories, Kumamoto, Japan) buffer (pH 5.4), EDC and sNHS were added to final concentrations of 2 and 5 mM, respectively. After 30 min of stirring, the pH of the WPU-MES buffer solution was adjusted to 7.4, and the peptide was added to this solution at 1 mg/mL and the mixture stirred for 24 h. In addition, the pH of the MES buffer was adjusted with sodium hydroxide (NaOH, Wako Pure Chemical Industries, Ltd., Tokyo, Japan). Following the completion of the cross-linking reaction, the solution was dialyzed against ultrapure water until the MES, EDC, sNHS, and unreacted peptide were removed. The final concentration of peptide modified WPU was ∼0.5% (w/v). Further, peptide modified WPU was named WPU-AAA and WPU-AYA based on the sequence of the peptide to be modified.

### 2.3 Fabrication of the SF/WPU Composite Nonwoven Sheet

For the material, a nonwoven sheet formed using the electrospinning (ESP) method was selected. The ESP method can electrically accumulate nano- to microorder fibers, and therefore, domain aggregation is suppressed in dissimilar polymer blend materials because of stretching during spinning and rapid solvent volatilization. This promotes compatibilization by refining the dispersed phase.^21,22^ ESP nonwoven fabric sheets were fabricated to maximize the effect of peptide modification by promoting compatibilization during the preparation of the SF/WPU composites.

The SF sponges and each type of WPU sponge were used to prepare the SF/WPU composite materials. Prior to preparing nonwoven sheets, the SF aqueous solution and WPU aqueous dispersion were processed into a sponge form. Each solution was adjusted to 1% (w/v) and freeze-dried for 72 h to obtain sponges.

Equal weights of the SF sponge and each type of WPU sponge were dissolved in 1,1,1,3,3,3-hexafluoro-isopropanol (HFIP, Tokyo Chemical Industry Co., Ltd., Tokyo, Japan) and blended at 7% (w/v). After 24 h of stirring, HFIP solutions filled in a syringe were electrospun from the tip of a needle attached to the syringe for 1.5 h under a laboratory condition (Fluidnatek LE-50, Bionicia, Spain).^23^ SF/WPU composite nonwoven sheets were fabricated by collecting the discharged solution on an aluminum foil attached to a collector 15 cm away. ESP conditions were standardized among samples and were set as follows: an inner needle diameter of 0.61 mm, applied voltage of 28 kV, and discharge rate of 1.3 mL/h. Nonwoven sheets were dried overnight and placed in 100% relative humidity for 2 h at 37 °C atmosphere to insolubilize them. The composite nonwoven sheets were named SW, SW-AAA, and SW-AYA based on the characteristics of the WPU.

### 2.4 Morphological Evaluation

The surface morphology of SF/WPU composite nonwoven sheets were evaluated with scanning electron microscopy (SEM) observations. The SEM observations were conducted using an ultradeep multiangle microscope VHX-D5100 (Keyence Corporation, Osaka, Japan) at 10 kV and ×3000 magnification. Observed images were analyzed by using the ImageJ open-source software to measure individual fiber diameters and angles against scale bars. The average fiber diameter and angular dispersion were calculated by performing the operation of measuring 40 randomly selected fibers thrice.

### 2.5 Structure Analysis

High-resolution solid-state ^13^C NMR spectra were acquired by using a Bruker DSX-400 AVANCE spectrometer employing CP/MAS with two-pulse phase modulation and ^1^H decoupling at 76 kHz. Pulse sequence parameters were set as follows: pulse delay time of 5 s, ^1^H 90° pulse of 3.25 μs, spinning rate of 8.5 kHz, and contact time of 2 ms. Further, ^13^C chemical shifts were referenced to tetramethylsilane using the methine carbon signal of adamantane at 29.47 ppm as an external standard. To evaluate crystallinity in the SF Ala C_β_ peak and the WPU–CH_2_–O- peak, waveform separation was performed using Origin2024b software, and the secondary structure percentage was calculated from the peak area ratio. The deconvolution plots created by waveform separation are shown in Figure S4-1∼8.

The ^1^H spin–lattice relaxation times in the laboratory frame (*T*_1_^H^) were indirectly measured from the well-resolved ^13^C signals enhanced by a CP of 2 ms applied after the saturation-recovery pulse sequence for ^1^H.^24^ The *T*_1_^H^ values were calculated using 3 points SF Ala C_β_ (19.5 ppm), Ala C_α_ (48.9 ppm), and Ser C_α_ (55.0 ppm) as SF components and 5 points –CH_2_–(25.3, 28.4, 31.6, 33.7 ppm), and –CH_2_–O- (64.9 ppm) from PCL, the PU main skeleton, as WPU components. Recovery time (τ) versus signal intensity (*M*_(*t*)_/*M*_(0)_) ratio was plotted and curve fitted using Origin 2024b software as an exponential function ( $\frac{M_{\left(t\right)}}{M_{\left(0\right)}}=1-e^{-t/{T_{1}}^{H}}$ ). The curve fitting results are shown in Figure S4-1∼8.

### 2.6 Mechanical Property Test

Mechanical properties of SF/WPU composite nonwoven sheets were evaluated using uniaxial tensile and dynamic mechanical analysis (DMA). Uniaxial tensile tests were performed by using an EZ test machine (Shimadzu Seisakusho Co. Ltd., Kyoto, Japan). The sample pieces were cut into JIS dumbbell-shaped No. 7 and immersed in ultrapure water for at least 24 h before measurement. The measurements were performed at a tensile speed of 5 mm/min with a 100 N load cell until the sample broke. Breaking stress, elongation, Young’s modulus, and fracture energy were calculated using the obtained stress–strain curves (*n* = 6). Young’s modulus was defined as the slope of the stress–strain curve in the 1–6% strain interval, and the fracture energy was defined as the area of the region bounded by the stress–stroke curve.

DMA measurements were performed using a DVA-205 instrument (IT Measurement Regulation Co., Ltd., Osaka, Japan). The sample pieces were 5 × 35 mm in width and length. The samples were heated from −100 to 300 °C with a heating rate of 2 °C/min to perform DMA measurement. The other measurement conditions were set as follows: tensile mode, tensile strain of 0.08%, and a frequency of 1 Hz. The glass transition points (*T*_g_) of SF and WPU and the crystal relaxation point (*T*_m_) of SF were read from the peak tops of the plot of the loss tangent (tan δ) versus temperature obtained from the measurements (*n* = 3).^25^

### 2.7 Statistical Analysis

All data were presented as the mean ± SD and analyzed by one-way analysis of variance (one-way ANOVA) followed by the Tukey’s multiple comparisons test. Statistical significance was considered at the *p* value < 0.01 vs SW, as shown with *.

## 3 Results

### 3.1 Morphology of SF/WPU Composite Nonwoven Sheets

Figure 1 shows SEM images of the SF/WPU composite nonwoven sheets. All sheets were composed of nanoscale fibers. Table 1 lists the average fiber diameter and angle to the scale bar of the composite nonwoven sheets. The average fiber diameter was over 600 nm for each sample, with no significant differences among samples. Further, no significant differences in the average fiber angle were observed among samples, and the standard deviation was sufficiently large relative to the average angle. The fiber angle here refers to the angle of each relative to the scale bar (the red bar in the image), which indicates the orientation of the fiber. These results confirm that nonwoven sheets were fabricated by laminating uniformly and randomly aligned fibers in all samples, with or without peptide modification.

**Figure 1:**
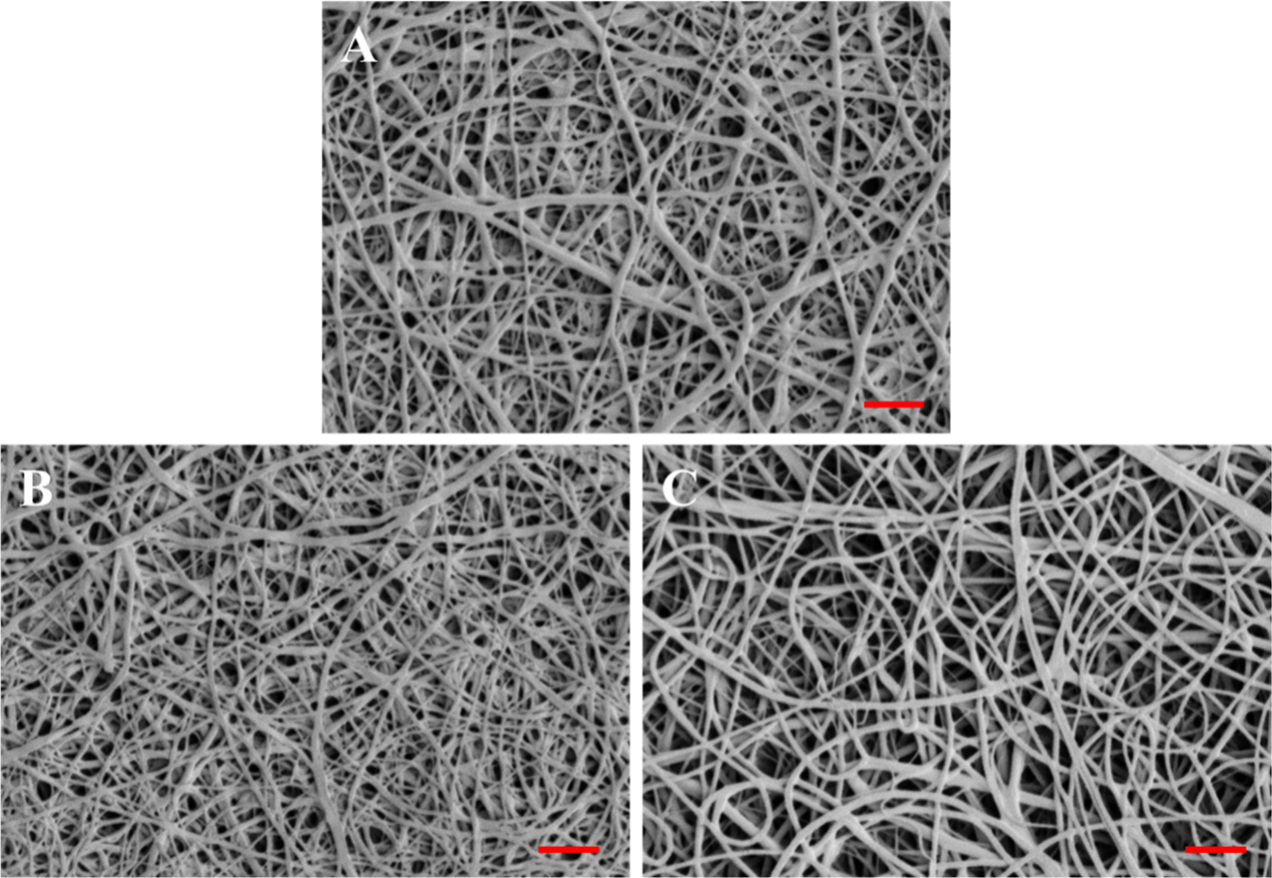
SEM images of (A) SW, (B) SW-AAA, and (C) SW-AYA composite nonwoven sheets (the red bars in the lower right of Figure 1A∼C indicate the scale bar, the width of which is 10 μm).

**Table 1:** Fiber Diameters and Angles of SF/WPU Composite Nonwoven Sheets

	SW	SW-AAA	SW-AYA
fiber diameter [nm]	658 ± 124	637 ± 101	627 ± 114
fiber angle [°]	–5.41 ± 42.1	–3.84 ± 40.2	5.55 ± 39.8

### 3.2 Structural Analysis of SF/WPU Composite Nonwoven Sheets

^13^C CP/MAS NMR measurements and ^1^H spin–lattice relaxation times (*T*_1_^H^) measurements were performed to analyze the structural properties of the SF/WPU composite nonwoven sheets. The ^13^C CP/MAS NMR spectra are shown in Figure 2 and the ^1^H spin–lattice relaxation times (*T*_1_^H^) are shown in Figure 3. The pure SF material evaluated in this study was in a nonwoven form, whereas the pure WPU material was in a film form because the low viscosity of WPU makes it difficult to form fibers on its own.

**Figure 2:**
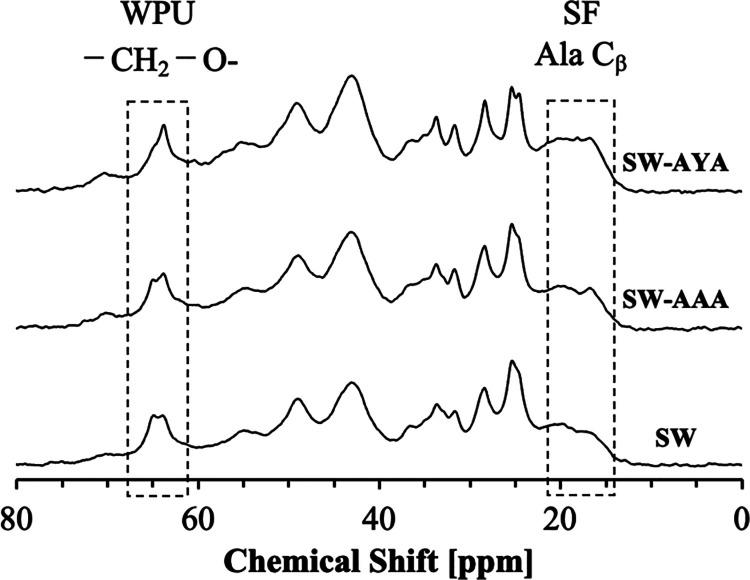
Expanded ^13^C CP/MAS NMR spectra of SF/WPU composite nonwoven sheets in the range of 0–80 ppm (full view of the spectra is shown in Figure S2).

**Figure 3:**
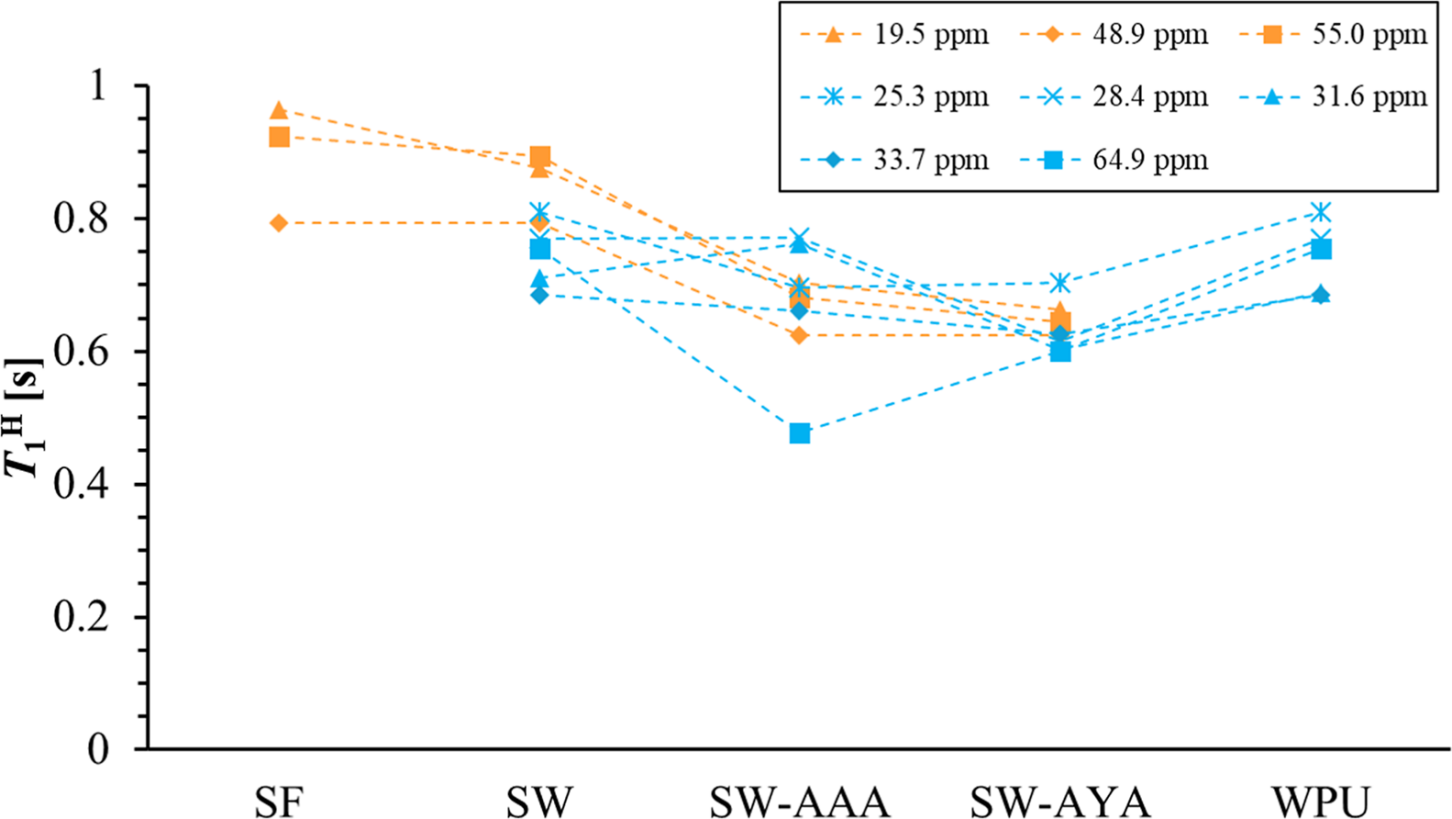
*T*_1_^H^ plot of SF (Ala C_β_: 19.5 ppm, Ala C_α_: 48.9 ppm, and Ser C_α_: 55.0 ppm) and WPU (–CH_2_–: 25.3, 28.4, 31.6, and 33.7 ppm; –CH_2_–O-: 64.9 ppm) in SF/WPU composite nonwoven sheets.

The characteristics peaks observed in SF and WPU were identified from the ^13^C CP/MAS NMR spectra of pure SF and WPU materials (Figure S1), respectively.^26^ Concretely, for SF, the peaks derived from the random coil and β-turn structures at 16.5 ppm and peaks derived from the β-sheet structures at 19.5 and 21.9 ppm were observed in the Ala C_β_ region. It is known that a peak derived from the β-sheet structure with alternating Ala side-chain methyl groups appears at 19.5 ppm, and a peak derived from the β-sheet structure with parallel Ala side-chain methyl groups appears at 21.9 ppm.^27^ Further, the Gly C_α_, Ala C_α_, and Ser C_α_ peaks at 43.5, 48.9, and 56.0 ppm, respectively, were observed. For WPU, the peak derived from the amorphous phase at 63.8 ppm, the peak derived from the interphase at 64.3 ppm, and the peak derived from the crystal phase at 64.9 ppm were identified in the –CH_2_–O- region of PCL, which is the main component of WPU.^28^ In addition, PCL –CH_2_– peaks were observed at 25.2, 28.9, 33.1, and 33.8 ppm.^29^ Further, these peaks were confirmed in the ^13^C CP/MAS NMR spectra (Figure 2) of the SF/WPU composite nonwoven sheets.

The proportional change in the components of the SF Ala C_β_ peaks (Table 2) revealed that the proportion of the peak at 16.5 ppm assigned to the random coil and repeating β-turn structure was slightly increased in SW-AAA. Also, in SW-AYA, the β-sheet structure with alternating Ala side-chain methyl groups, which is attributed at 19.5 ppm, accounts for a larger percentage of the total β-sheet structure than in the other samples.

**Table 2:** Ratio of Random Coil and Repeated β-turn (16.5 ppm) and β-sheet (19.5, 21.9 ppm) of SF/WPU Composite Nonwoven Sheets (Determined by the Deconvolution of the SF Ala C_β_ Peaks)

chemical shifts of the Ala C_β_ peak in SF (structure which was assigned to)	SW	SW-AAA	SW-AYA
16.5 ppm (random coil and repeated β turn)	36	40	36
19.5 ppm (β sheet)	19	14	25
21.9 (β sheet)	45	45	39

The proportion change in the components of the WPU–CH_2_–O- peaks (Table 3) revealed that the proportion of the peak at 63.8 ppm assigned to the amorphous phase was increased by peptide modification, especially in SW-AYA. Correspondingly, the proportion of the peak at 64.9 ppm assigned to the crystal phase decreased. The proportion of the peak at 64.3 ppm attributed to the interphase showed increasing trends similar to those of the amorphous phase.

**Table 3:** Ratio of the Amorphous Phase (63.8 ppm), Interphase (64.3 ppm), and Crystal Phase (64.9 ppm) of SF/WPU Composite Nonwoven Sheets Determined by the Deconvolution of the WPU–CH_2_–O- Peaks

chemical shifts of –CH_2_–O- peak in WPU (structure which was assigned to)	SW	SW-AAA	SW-AYA
63.8 ppm (amorphous phase)	58	69	77
64.3 ppm (interphase)	2	3	6
64.9 ppm (crystal phase)	40	29	16

Figure 3 shows ^1^H spin–lattice relaxation times (*T*_1_^H^) of SF/WPU composite nonwoven sheets, pure SF, and pure WPU. Dipole interactions cause an exchange of energy between ^1^H spins inherent in SF and WPU when the SF and WPU domains are close together (within a few tens of nanometers).^30^ This averages the difference in relaxation times and shows the same relaxation time for SF and WPU. *T*_1_^H^ values were calculated from peaks attributed to Ala C_β_, Ala C_α_, and Ser C_α_ at 19.5, 48.9, and 55.0 ppm, respectively, in SF, and from peaks attributed to –CH_2_– at 25.3, 28.4, 31.6, and 33.7 ppm and –CH_2_–O- at 64.9 ppm. The *T*_1_^H^ value of pure SF was ∼0.89 s, and that of pure WPU was ∼0.74 s. The *T*_1_^H^ values of SF and WPU remained almost unchanged in SW, whereas they were altered in peptide-modified samples. In SW-AAA, the *T*_1_^H^ values for SF and WPU were ∼0.67 and 0.67 s, respectively, and in SW-AYA, the *T*_1_^H^ values for SF and WPU were ∼0.64 and 0.63 s, respectively. These results indicate that *T*_1_^H^ values in both SF and WPU domains were not changed by simply mixing SF and WPU; however, they were significantly decreased and averaged by modifying the peptides.

### 3.3 Mechanical Properties of the SF/WPU Composite Nonwoven Sheets

Figure 4 shows the stress–strain curves of SF/WPU composite nonwoven sheets. The curve shapes differed among the samples. Physical property parameters calculated from the stress–strain curves are shown in Figure 5, where (A), (B), (C), and (D) represent the maximum stress, breaking elongation, Young’s modulus, and fracture energy, respectively. These four mechanical property parameters were higher after peptide modification for SW-AAA.

**Figure 4:**
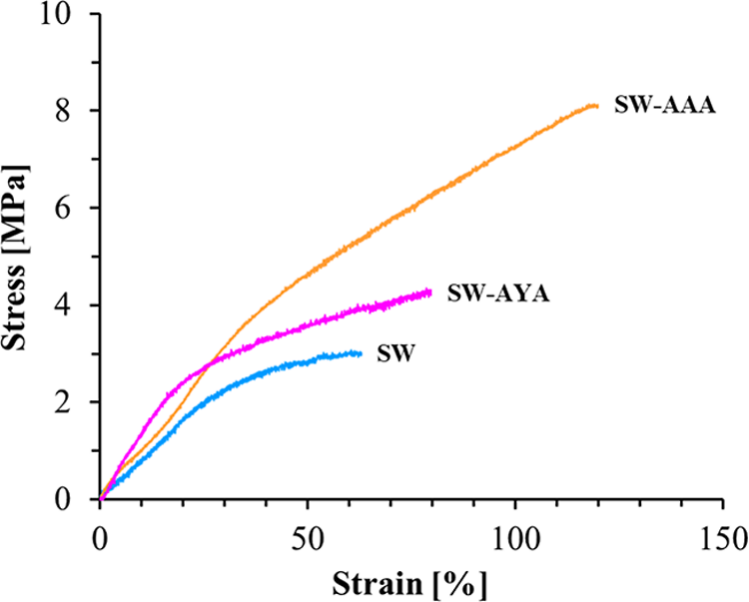
Stress–strain curve of SF/WPU composite nonwoven sheets in the wet state.

**Figure 5:**
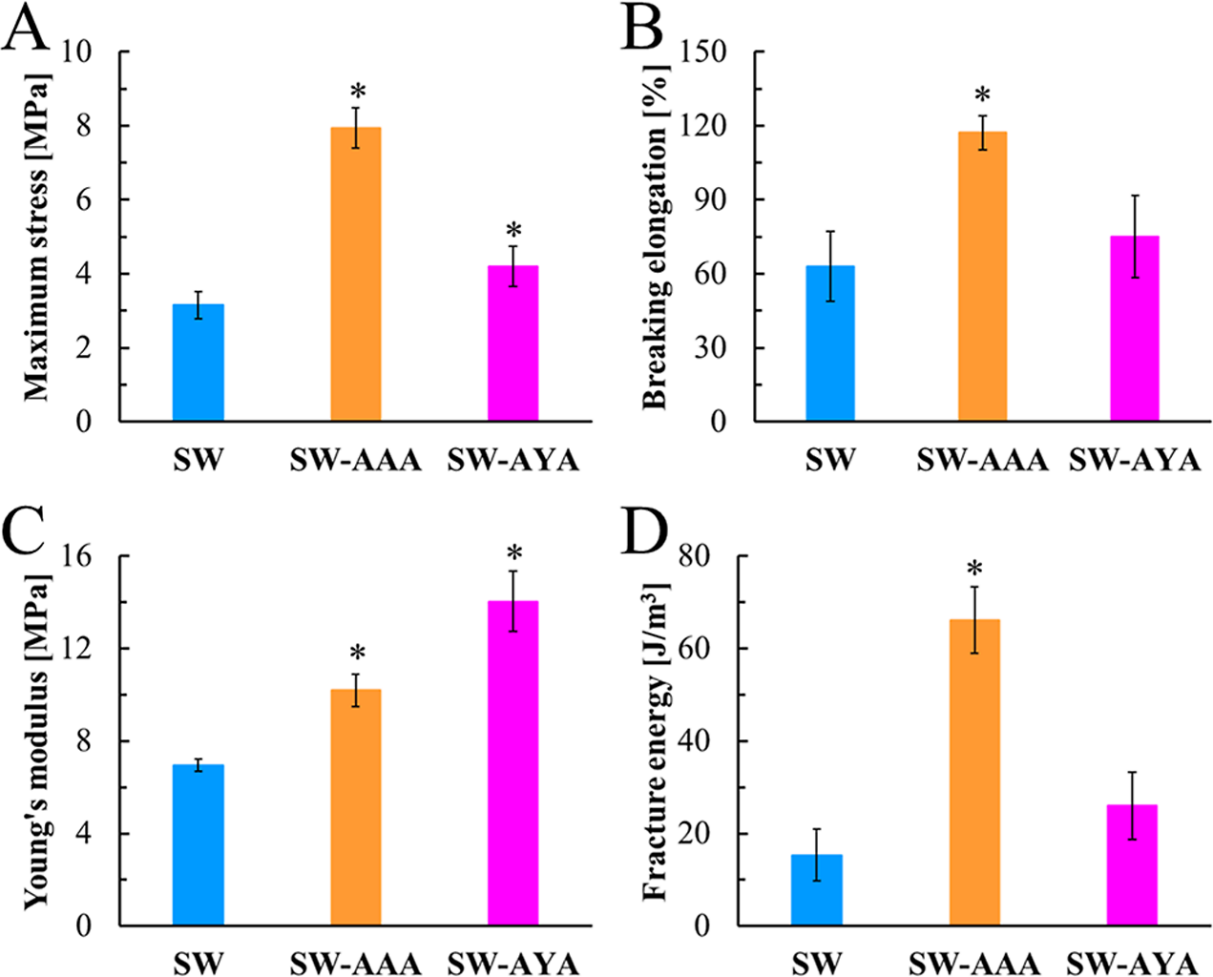
Mechanical property parameters calculated by the stress–strain curve: (A) maximum stress, (B) breaking elongation, (C) Young’s modulus, and (D) fracture energy.

Figure 6 shows the loss tangent (tan δ) versus temperature for SF/WPU composite nonwoven sheets. The tan δ value, which is an indicator of the viscoelasticity of a material, was almost the same throughout the measurement range for all of the samples. Further, the glass transition point (*T*_g_) and crystal relaxation point (*T*_m_) were obtained from peaks in the tan δ plot. The peaks at ∼60, 200, and 270 °C were assigned to *T*_g_ of WPU,^31^ *T*_g_ of SF,^32^ and *T*_m_ of SF,^33^ respectively, using the previous paper as a reference. These peaks were identified in all of the samples. Table 4 lists the *T*_g_ values of WPU and SF and the *T*_m_ values of SF for each sample. The *T*_g_ value of WPU shifted by ∼6 °C higher, whereas the *T*_g_ value of SF shifted by ∼1–2 °C lower after peptide modification. The *T*_m_ value of SF shifted by more than 10 °C after peptide modification. Furthermore, shifts in *T*_g_ and *T*_m_ were observed for SW-AAA that were larger than those for SW-AYA.

**Figure 6:**
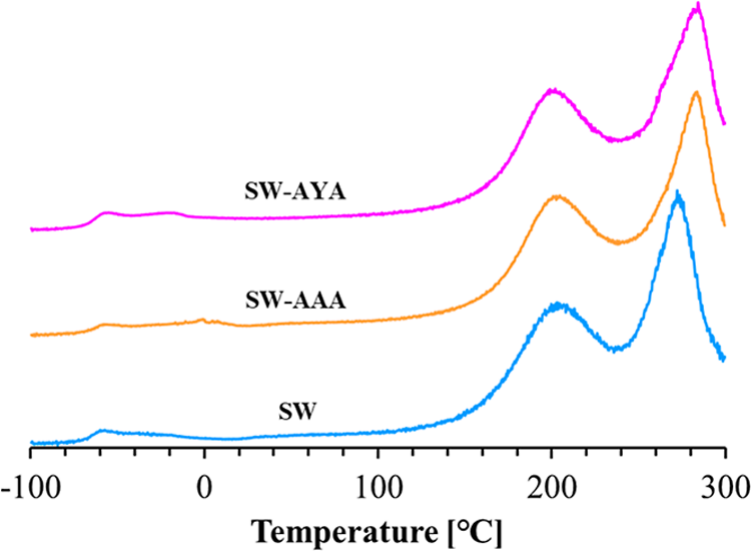
Temperature dependence of the loss tangent (tan δ) of SF/WPU composite nonwoven sheets.

**Table 4:** Glass Transition Point (*T*_g_) and Crystal Relaxation Point (*T*_m_) Read from a tan δ Curve

	SW	SW-AAA	SW-AYA
*T*_g_ of WPU [°C]	–58.5 ± 1.58	–52.1 ± 1.00	–52.9 ± 2.32
*T*_g_ of SF [°C]	204 ± 1.22	202 ± 0.276 *	203 ± 0.962
*T*_m_ of SF [°C]	272 ± 0.187	284 ± 1.58 *	283 ± 1.19 *

## 4 Discussion

Despite the fact that the lack of miscibility has been observed to degrade material strength during the fabrication of SF/polymer composite materials, this study aims to propose a new method for improving the miscibility of SF/polymer composite materials. In existing research on improving the miscibility of this material, methods such as the chemical modification of SF to form cross-linking points and solvent optimization have been tried. However, these methods are limited to specific types of polymers and may significantly alter the characteristic structure of SF, thereby impairing its properties. An interaction between the SF and the polymer is formed by modifying the polymer with (GX)_*n*_ motif peptides. Further, we hypothesized that the formation of this interaction would improve interfacial adhesion and the miscibility of SF/polymer composite materials. The physical properties and structures of the fabricated SF/WPU composite materials were analyzed to test this hypothesis.

The material morphology analysis using SEM confirmed that nanoorder fibers were laminated in all SF/WPU composite nonwoven sheets. No significant differences were observed between samples in terms of the average fiber diameter and average fiber angle. The fiber diameter and orientation have a significant effect on the material properties of nonwoven sheet materials. Therefore, physical property changes between samples depend only on the sample properties in terms of material morphology.

In the material structure analysis by solid-state ^13^C NMR from CP/MAS measurements, the spectral shape of the SF/WPU composite nonwoven sheets changed before and after peptide modification. Significant changes are observed in the SF Ala C_β_ and WPU–CH_2_–O- peaks. Both peaks were derived from the crystalline and amorphous structures. The crystallinity of the materials was analyzed by calculating the intensity ratios using peak deconvolution. In the SF Ala C_β_ peak, SW-AAA showed a slight increase in the peak fraction at 16.5 ppm, which confirmed a decrease in the crystallinity of the SF domain. The β-sheet structure percentage ratios at 19.5 and 21.9 ppm were significantly different when compared with samples modified with different peptides. This result indicates that the introduction of Tyr changed the packing pattern in SF; however, the direct reason for this fine structural change and its effect on the physical properties are unknown. In WPU, the –CH_2_–O- peak of the peptide-modified samples exhibited a lower peak fraction (64.9 ppm), which confirms reduced crystallinity in the WPU domain. The peak percentage increased in SW-AYA at 63.8 and 64.3 ppm by comparing samples modified with different peptides, which indicates that the crystal structure of WPU was dynamically changed by Tyr introduction. This can be attributed to the high steric hindrance of the Tyr side chain, physically inhibiting the formation of the crystal structure of WPU by forming domains through the binding of peptides and SF molecules to each other. From *T*_1_^H^ measurements, the molecular mobilities of both SF and WPU domains of SF/WPU composite nonwoven sheets changed before and after peptide modification. In particular, the change in the *T*_1_^H^ value of the WPU-derived 64.9 ppm peak was characteristic for each sample: for SW, the value was about 0.75 s, which is almost identical to that of pure WPU, indicating that the kinetics of WPU was not changed by mixing with SF. On the other hand, the values for SW-AAA and SW-AYA were about 0.48 and 0.60 s, respectively, which were significantly lower than those for SW. The decrease in *T*_1_^H^ values indicates an increase in molecular mobility, and the 64.9 ppm peak is attributed to the –CH_2_–O- crystal structure itself. *T*_1_^H^ value in the 64.9 ppm peak for SW-AAA compared to SW-AYA, which could be related to the results for breaking elongation in the tensile test. However, so far, the detailed reason for the significant change in mobility between SW-AAA and SW-AYA is unclear. One possibility is that by introducing Tyr into the peptide sequence, the Tyr of the peptide and the Tyr of the SF molecular chain interact due to π–π stacking. This interaction could result in reduced mobility in AYA compared to that in AAA. Therefore, in future, we plan to elucidate a mobility change using peptides with amino acid residues and aromatic rings in the side chain, as well as Tyr. Subsequently, the average *T*_1_^H^ values were evaluated with reference to previous reports.^32^ The *T*_1_^H^ value of the SF-derived peak decreased from about 0.89 to about 0.85 s as the WPU was mixed in. On the other hand, the *T*_1_^H^ value of the WPU-derived peak hardly changed from about 0.74 to about 0.75 s as SF was mixed. This indicates that the mobility of SF was enhanced by mixing with WPU, whereas that of WPU was not changed by mixing with SF. In contrast, the *T*_1_^H^ values of the SF-derived and WPU-derived peaks in SW-AAA were about 0.67 and 0.67 s, respectively. This indicates that the *T*_1_^H^ values of both domains were reduced compared to those of SW, indicating improved mobility. This result is consistent with the crystal structure percentage calculation in the solid-state ^13^C CP/MAS NMR measurements. The difference in the *T*_1_^H^ values for both domains is also smaller. It is known that in polymer blend materials, when polymers with different relaxation times are in close proximity to each other, the difference in relaxation times is averaged by spin diffusion between the polymers due to the dipole interaction between the very strong proton nuclei. Therefore, the modification of the AAA peptide improved the miscibility of the SF/WPU composites: in SW-AYA, the *T*_1_^H^ values of the SF-derived peak were about 0.64 s and that of the WPU-derived peak was about 0.63 s. This indicates increased mobility in both domains, which is consistent with the results of the crystal structure fraction calculations in the solid-state ^13^C CP/MAS NMR measurements. In SW-AYA, as in SW-AAA, the proximity of the *T*_1_^H^ values of both domains was also observed, confirming the improved miscibility. The closer proximity of SW-AAA indicates that the miscibility improvement effect is greater for AAA than for AYA, which is consistent with the discussion of the miscibility improvement effect in terms of thermal properties discussed below.

The stress–strain curves of the SF/WPU composite nonwoven sheets changed before and after peptide modification in the physical property analysis. The maximum stress, breaking elongation, Young’s modulus, and fracture energy calculated from the curves were enhanced for peptide-modified samples. This implies that the material acquired load resistance, ductility, stiffness, and toughness. Compared with samples modified with different peptides, they were markedly improved in SW-AAA. Also, it is interesting to note that only Young’s modulus shows a different trend from the other property parameters, with SW-AYA showing a higher value than SW-AAA. This is likely due to the π–π stacking of the aromatic rings of Tyr in the peptides modified with SF and WPU, resulting in higher stiffness in the early tensile phase.^34^ In the late tensile phase, when large tensile stresses are conserved, the higher steric hindrance of Tyr may have prevented optimization through reorientation of the molecular structure, resulting in greater SW-AAA for breaking elongation and maximum stress. These considerations need to be verified by structural analysis during tensile deformation using in situ X-ray diffraction (XRD) measurements and for samples after tensile testing. These results are consistent with the fact that peptide modification reduces the crystallinity of both the SF and WPU domains and improves miscibility in terms of the relaxation time. From DMA measurements, the plot of tan δ versus the temperature shift of SF/WPU composite nonwoven sheets changed before and after peptide modification. For *T*_g_ of SF and WPU and *T*_m_ of SF, a peak shift caused by peptide modification was observed. The peptide modification of the material shifted the *T*_g_ values of WPU and SF to higher and lower temperatures, respectively. In two polymer blend materials, the proximity of the *T*_g_ values of both domains suggests improved miscibility.^35^ Therefore, this result indicates that the peptide modification of the SF/WPU composite nonwoven sheet improved the miscibility of SF and WPU in terms of thermal properties. Between samples modified with different peptides, both *T*_g_ values tended to be closer for SW-AAA, which suggests that the miscibility improvement effect was greater with the modified AAA peptide than with the modified AYA peptide. In addition, the *T*_m_ of SF shifted to higher temperatures upon peptide modification, which implies that the thermal stability of the crystalline component of SF was improved by peptide modification. Compared with SW-AYA, SW-AAA showed a higher temperature shift in the *T*_m_ value of SF, and modification of the AAA peptide provided higher thermal stability to the material.

The surface morphology of the SF/WPU composite nonwoven sheets was not changed by the peptide modification of the polymer. Further, the miscibility between SF and WPU was demonstrated in terms of the molecular mobility and thermal properties, and the crystallinity was found to decrease in both the SF and WPU domains. These can be considered in relation to changes in physical properties, which indicate the possibility that peptide modification can precisely control the physical properties, and the molecular structure of composite sheets. In the future, the consistency of results obtained in this study should be investigated using peptides with adjusted sequence lengths and amino acid residues. Further structural analysis is required for understanding the relationship between the structural and physical properties.

## 5 Conclusion

We focused on degrading the material strength caused by the low compatibility of SF/polymer composite materials. The SF primary structure motif peptides were modified into WPU, and the physical and structural properties of SF/WPU composite nonwoven sheets were evaluated. These tests confirmed the effects of peptide modification.

From CP/MAS analysis using solid-state ^13^C NMR, the crystal structure proportions of SF and WPU were calculated, indicating that the crystallinity decreased after peptide modification. *T*_1_^H^ analysis also showed that the peptide modification improved the mobility of both domains as well as the miscibility. It was also found that AAA peptides were more effective in improving miscibility than AYA peptides. In the tensile test, peptide modification improved physical properties, especially the significant improvement in the AAA peptide-modified sample, which is consistent with the structural findings. In DMA measurements, the glass transition temperatures of both domains were close after peptide modification, with the highest proximity observed in the AAA peptide-modified sample. This indicates that peptide modification improves miscibility in terms of thermal properties, especially for the AAA peptide. The findings from the thermal property analysis were consistent with the structure findings.

We successfully evaluated the effects of peptide modification on the physical properties and structural changes of SF/WPU composite nonwoven sheets. These results suggest that this is an innovative method for improving the miscibility of SF-polymer composite materials in a simpler and more versatile approach than the existing methods.

## Supplementary Material



## Notes

^13^C CP/MAS NMR spectra of pure SF and WPU and SF/WPU composite nonwoven sheets; peak deconvolution in SF Ala C_β_ and WPU–CH_2_–O- peaks of the ^13^C CP/MAS NMR spectra; and peak intensity versus variable delay time and curve fitting results (PDF)

## Abbreviations

SF: silk fibroin
Gly: glycine
Ala: alanine
Ser: serine
WPU: water-dispersed polyurethane
ESP: electrospinning
CP/MAS: cross-polarization magic angle spinning
^13^C NMR: carbon-13 nuclear magnetic resonance
LiBr: lithium bromide
EDC: *N*-(3-(dimethylamino)­propyl)-*N*′-ethylcarbodiimide hydrochloride
sNHS: sulfo *N*-hydroxysuccinimide
MES: 2-morpholinoethanesulfonic acid
HFIP: 1,1,1,3,3,3-hexafluoro-isopropanol
SEM: scanning electron microscopy
DMA: dynamic mechanical analysis
