# Supplementary material for: Silk-Derived Peptide Modification to Polymers Improves the Miscibility of Composite Materials with Silk Fibroin
Source: ACS Omega. 2025 Oct 24;10(43):50995–1003. doi: 10.1021/acsomega.5c05282 (PMC12593957; doi:10.1021/acsomega.5c05282)
Supplement: Supplementary file 1 [file ao5c05282_si_001.pdf]

SUPPLEMENTARY INFORMATION

Silk-Derived Peptide Modification to Polymers Improves Miscibility of Composite Materials with Silk Fibroin

Yuri MATSUMOTO[1], *Shota AKIOKA*[1], *Yasumoto NAKAZAWA*[1]*

[1] Department of Biotechnology and Life Science, Tokyo University of Agriculture and Technology, 2-24-16 Naka-cho, Tokyo 184-8588, Japan

*Email: y-nakazawa@go.tuat.ac.jp

SUPPLEMENTARY INFORMATION

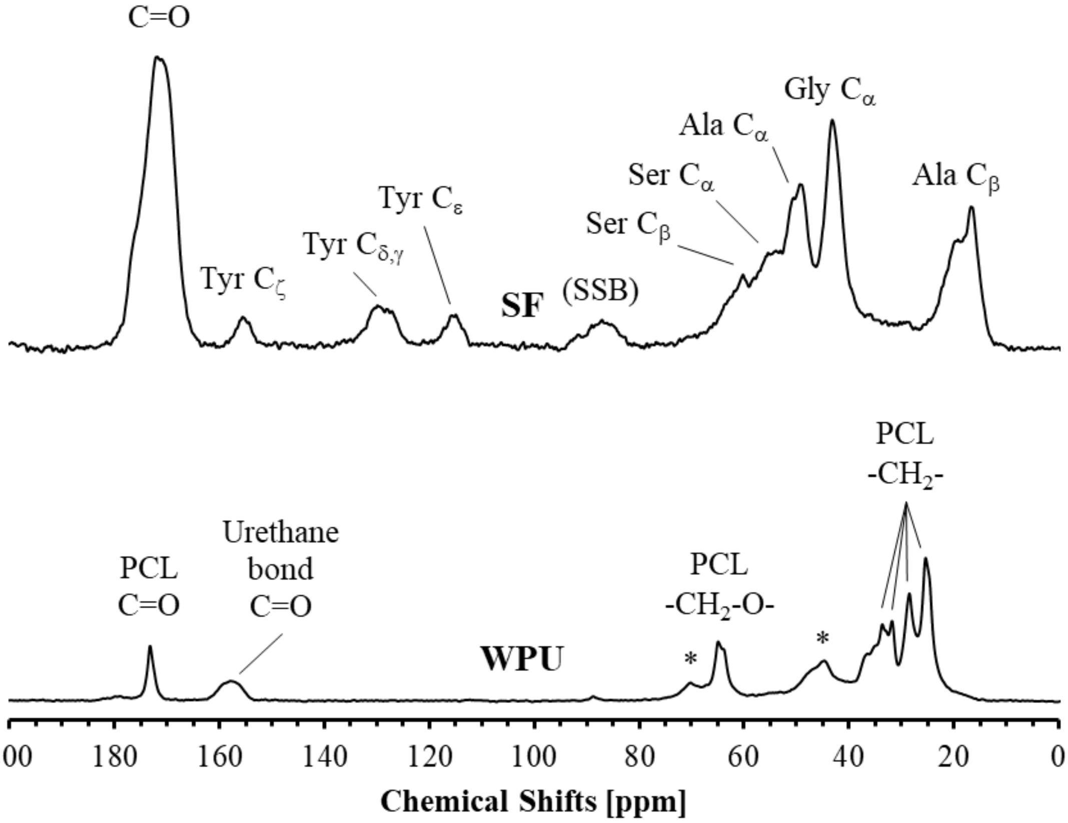

Figure.S1 ^{13}C CP/MAS NMR spectra of pure SF and WPU materials and attribution of each peak.

(* in the WPU structure cannot be disclosed for confidentiality reasons)

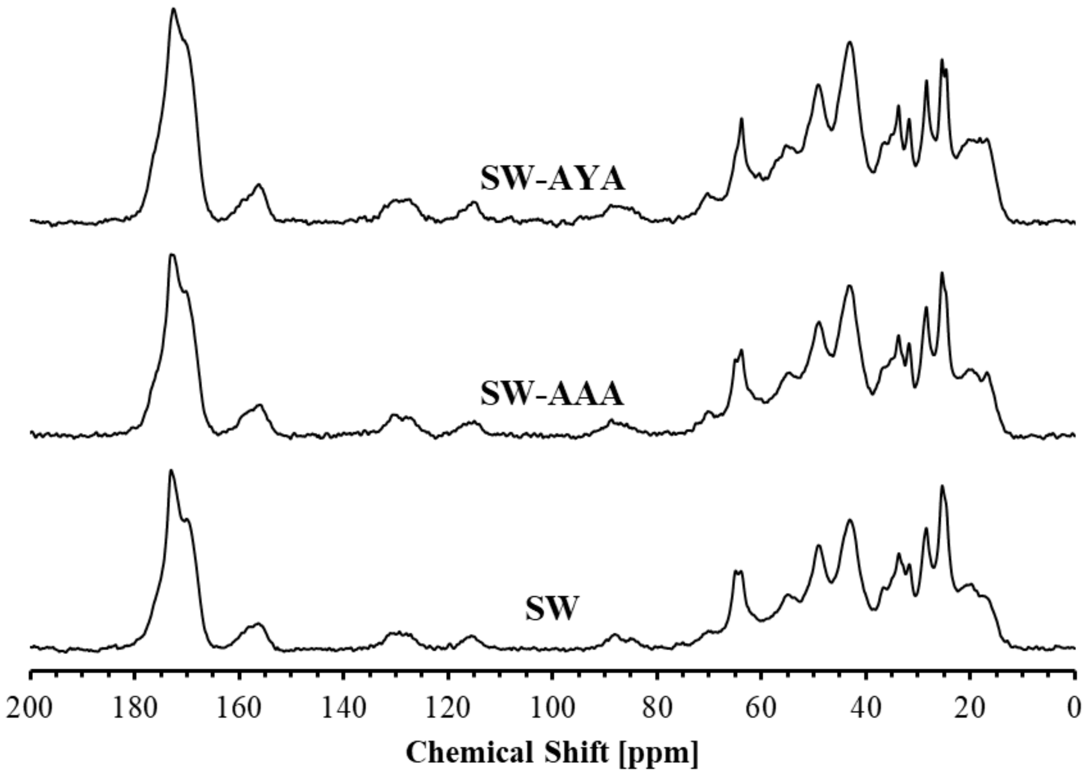

Figure.S2 ^{13}C CP/MAS NMR full view of spectra of SF/WPU composite non-woven sheets.

SUPPLEMENTARY INFORMATION

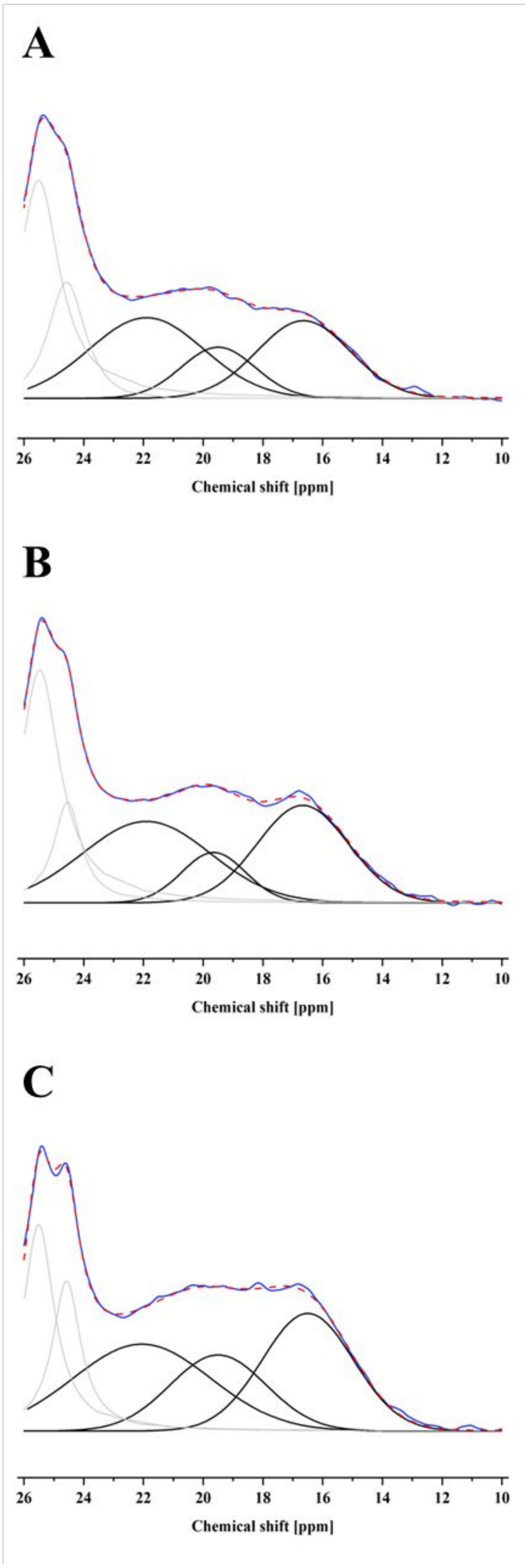

SUPPLEMENTARY INFORMATION

Figure.S3-1 Peak deconvolution in SF Ala C_β peak of the ^{13}C CP/MAS NMR spectra for SF/WPU composite non-woven sheets.

A) SW, B) SW-AAA, C) SW-AYA

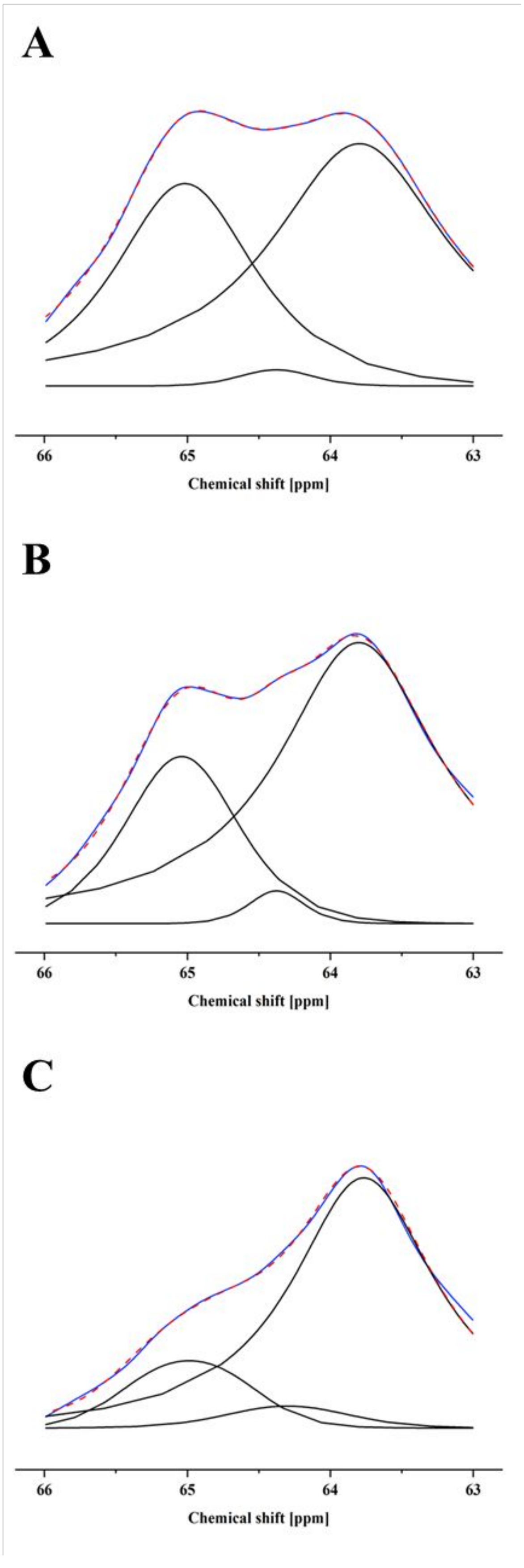
A
66
65
64
63
Chemical shift [ppm]
B
66
65
64
63
Chemical shift [ppm]
C
66
65
64
63
Chemical shift [ppm]

Figure.S3-2 Peak deconvolution in WPU -CH_2-O- peak of the ^{13}C CP/MAS NMR spectra for SF/WPU composite non-woven sheets.

A) SW, B) SW-AAA, C) SW-AYA

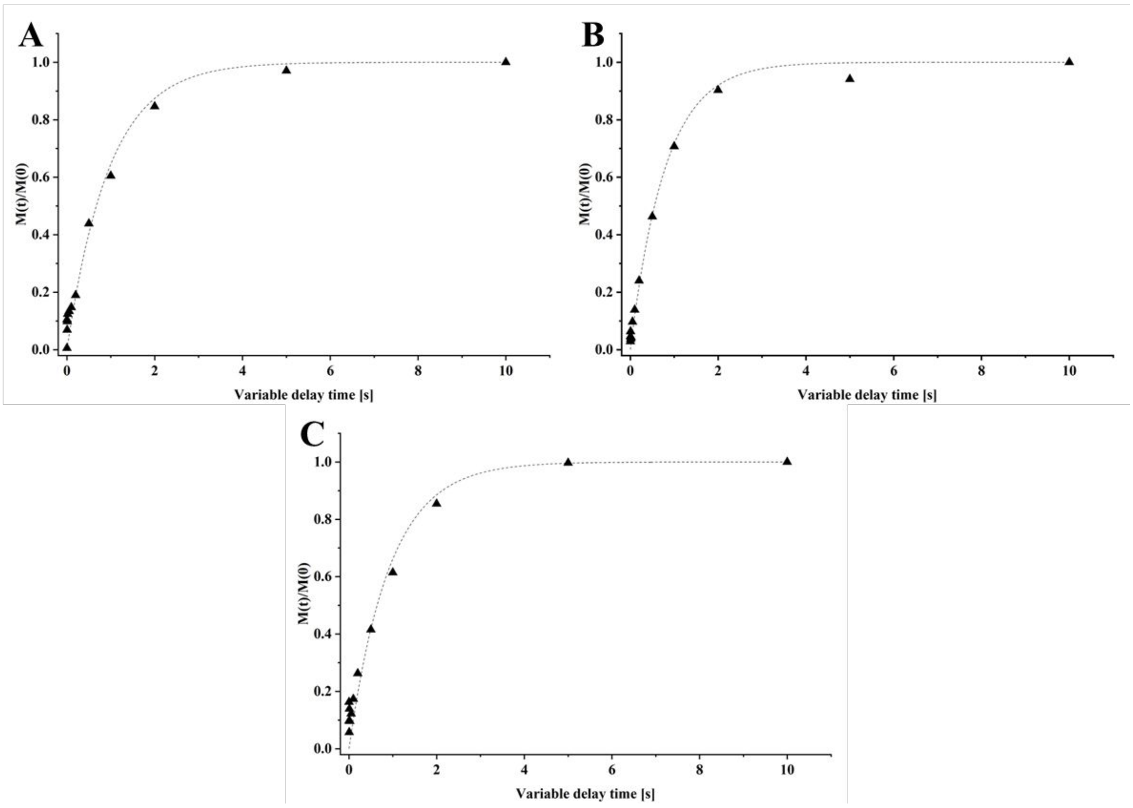

Figure.S4-1 The plot obtained from peak intensity versus variable delay time and fitting curve simulated from $M_{(t)}/M_{(0)} = 1 - e^{-\frac{t}{T_1^{\mathrm{H}}}}$ of SF.

A) Ala C_β (19.5 ppm), B) Ala C_α (48.9 ppm), C) Ser C_α (55.0 ppm)

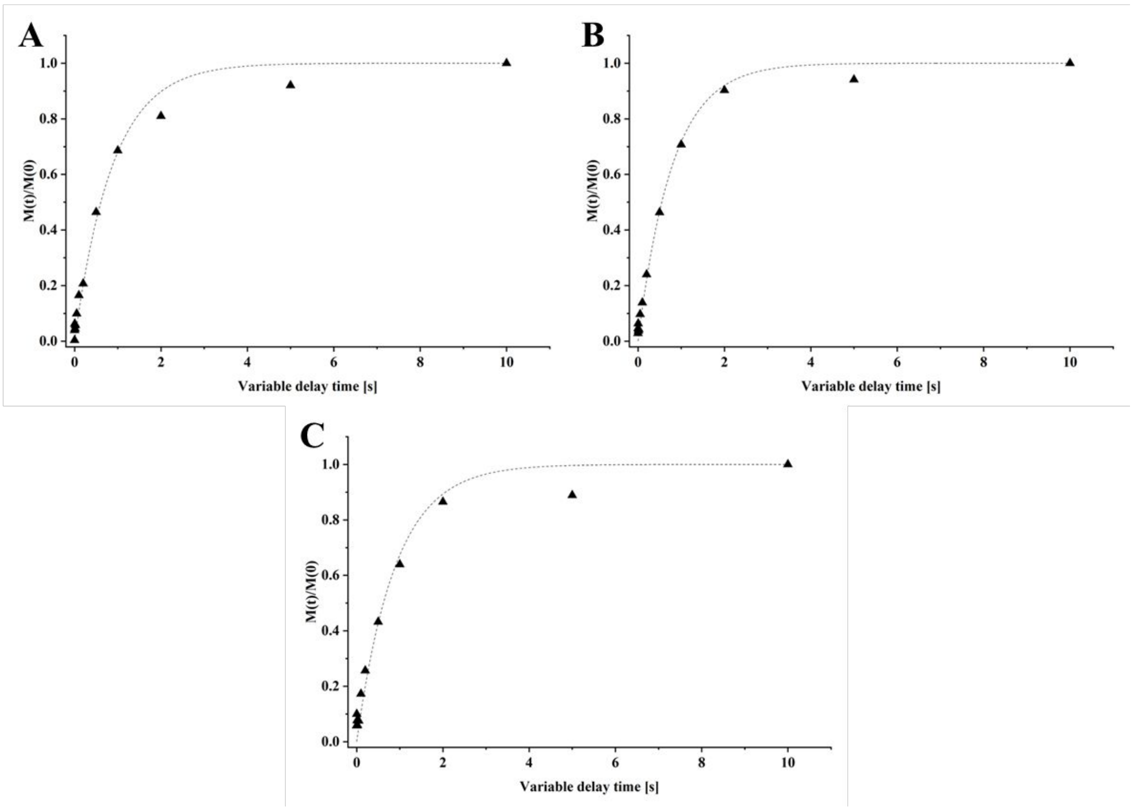

Figure.S4-2 The plot obtained from peak intensity versus variable delay time and fitting curve simulated from $M_{(t)}/M_{(0)} = 1 - e^{-\frac{t}{T_1^{\mathrm{H}}}}$ of SW.

A) Ala C_β (19.5 ppm), B) Ala C_α (48.9 ppm), C) Ser C_α (55.0 ppm).

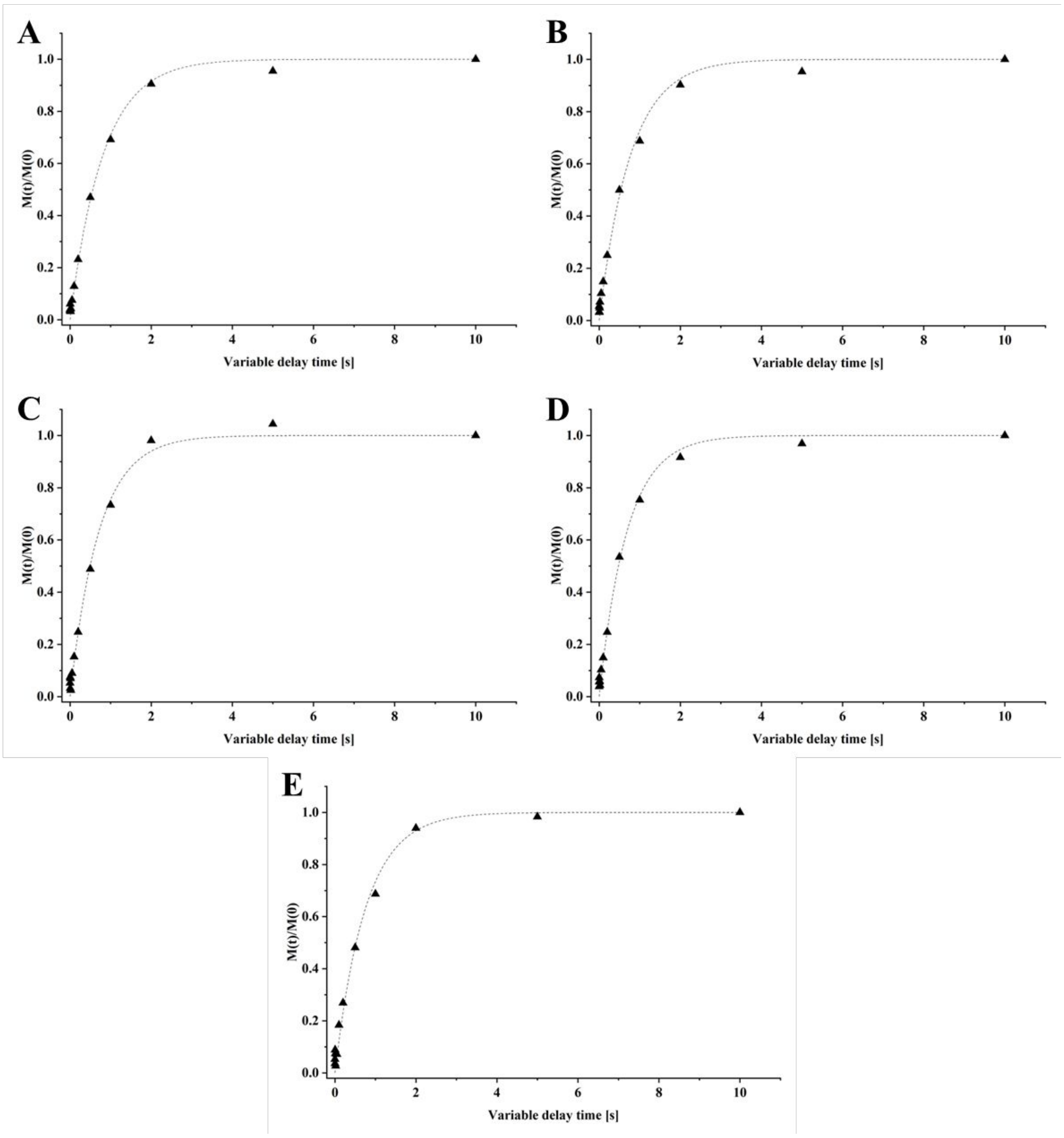

Figure.S4-3 The plot obtained from peak intensity versus variable delay time and fitting curve simulated from $M_{(t)}/M_{(0)} = 1 - e^{-\frac{t}{T_1^{\mathrm{H}}}}$ of SW.

A) WPU $-CH_2-$ (25.3 ppm), B) WPU $-CH_2-$ (28.4 ppm), C) WPU $-CH_2-$ (31.6 ppm), D) WPU $-CH_2-$ (33.7 ppm), E) WPU $-CH_2$-O- (64.9 ppm).

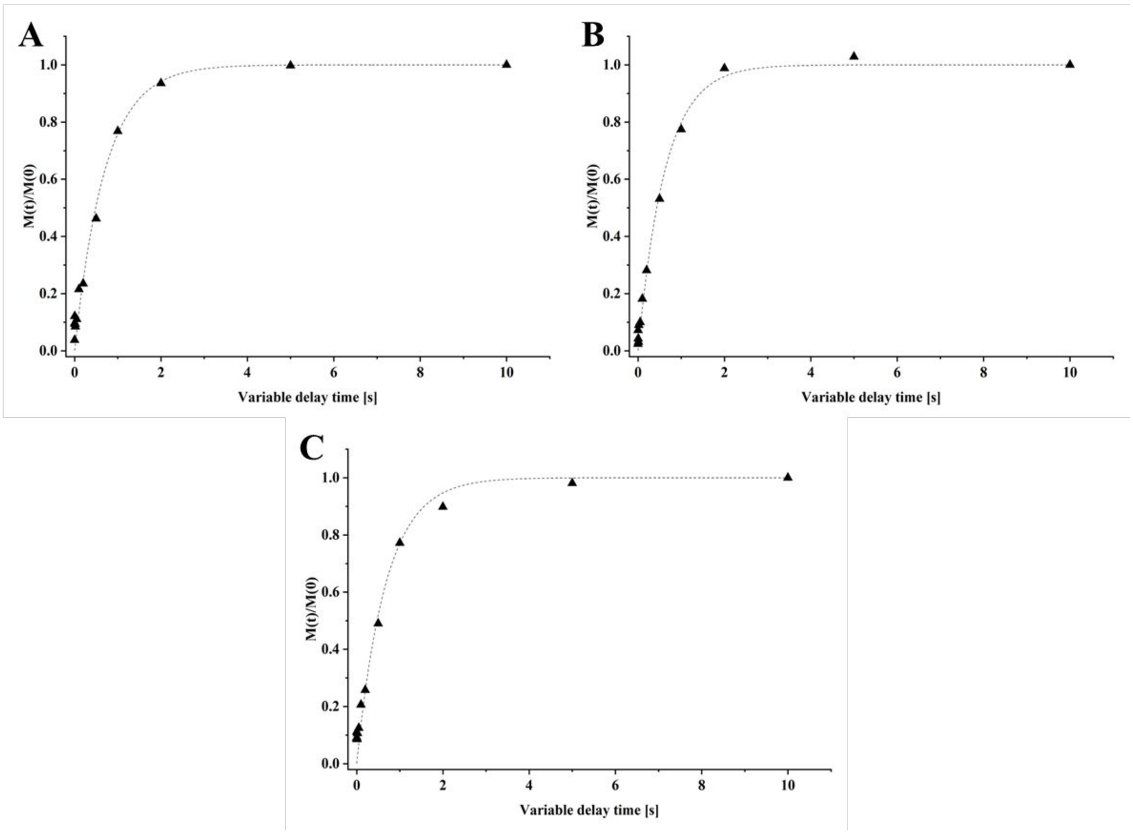

Figure.S4-4 The plot obtained from peak intensity versus variable delay time and fitting curve simulated from $M_{(t)}/M_{(0)} = 1 - e^{-\frac{t}{T_1^{\mathrm{H}}}}$ of SW-AAA.

A) Ala C_β (19.5 ppm), B) Ala C_α (48.9 ppm), C) Ser C_α (55.0 ppm).

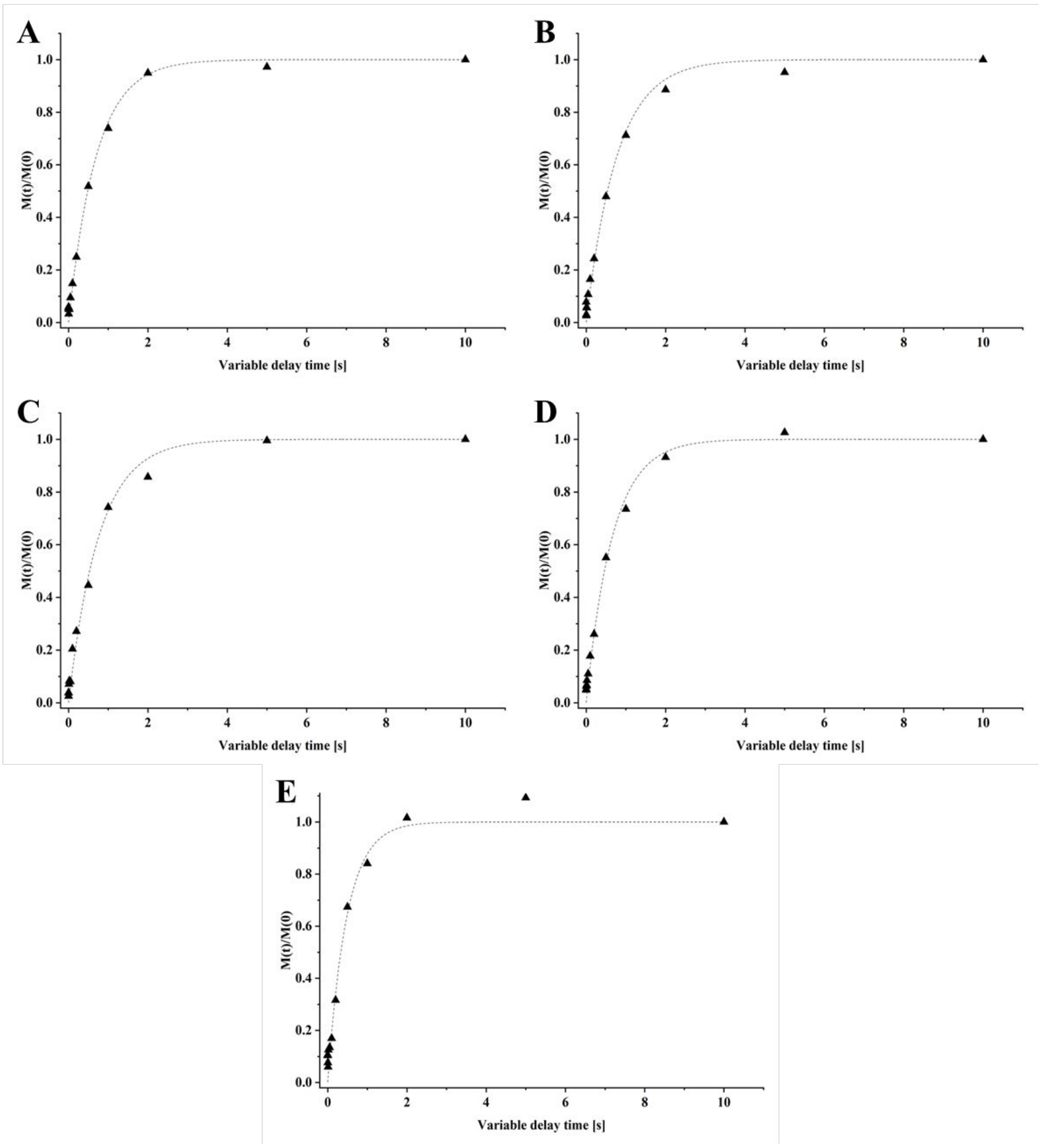

Figure.S4-5 The plot obtained from peak intensity versus variable delay time and fitting curve simulated from $M_{(t)}/M_{(0)} = 1 - e^{-\frac{t}{T_1^{\mathrm{H}}}}$ of SW-AAA.

A) WPU -CH_2- (25.3 ppm), B) WPU -CH_2- (28.4 ppm), C) WPU -CH_2- (31.6 ppm),

D) WPU -CH_2- (33.7 ppm), E) WPU -CH_2-O- (64.9 ppm).

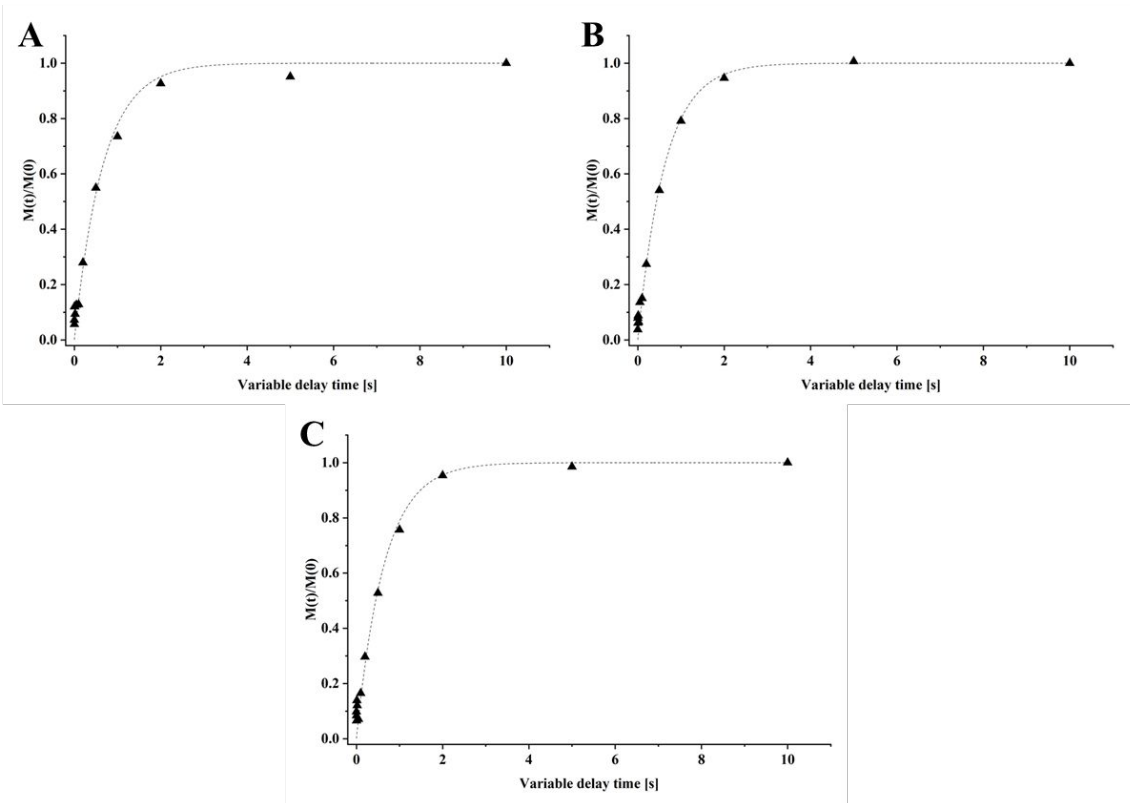

Figure.S4-6 The plot obtained from peak intensity versus variable delay time and fitting curve simulated from $M_{(t)}/M_{(0)} = 1 - e^{-\frac{t}{T_1^{\mathrm{H}}}}$ of SW-AYA.

A) Ala C_β (19.5 ppm), B) Ala C_α (48.9 ppm), C) Ser C_α (55.0 ppm).

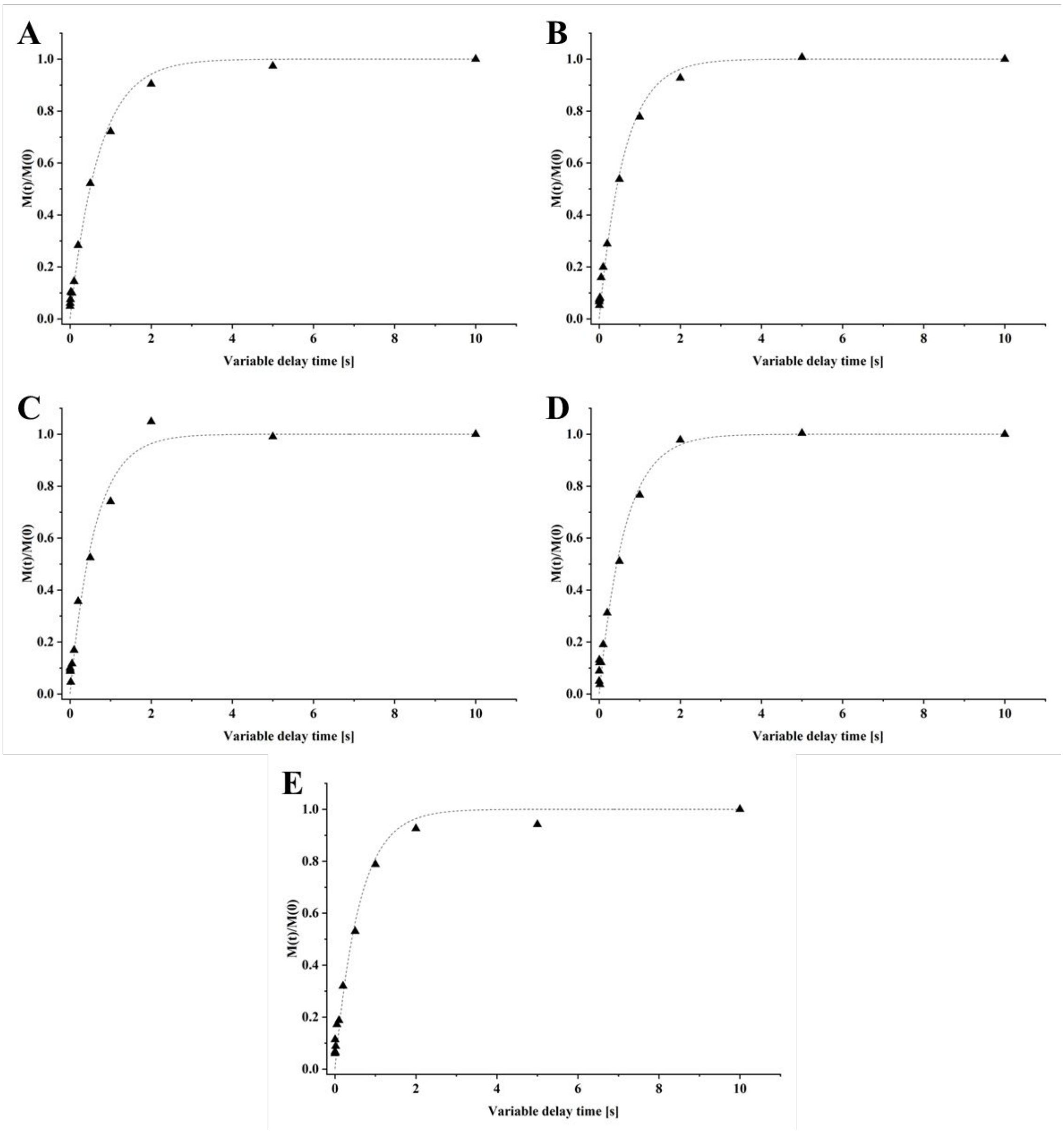

Figure.S4-7 The plot obtained from peak intensity versus variable delay time and fitting curve simulated from $M_{(t)}/M_{(0)} = 1 - e^{-\frac{t}{T_1^{\mathrm{H}}}}$ of SW-AYA.

A) WPU $-CH_2-$ (25.3 ppm), B) WPU $-CH_2-$ (28.4 ppm), C) WPU $-CH_2-$ (31.6 ppm), D) WPU $-CH_2-$ (33.7 ppm), E) WPU $-CH_2-O-$ (64.9 ppm).

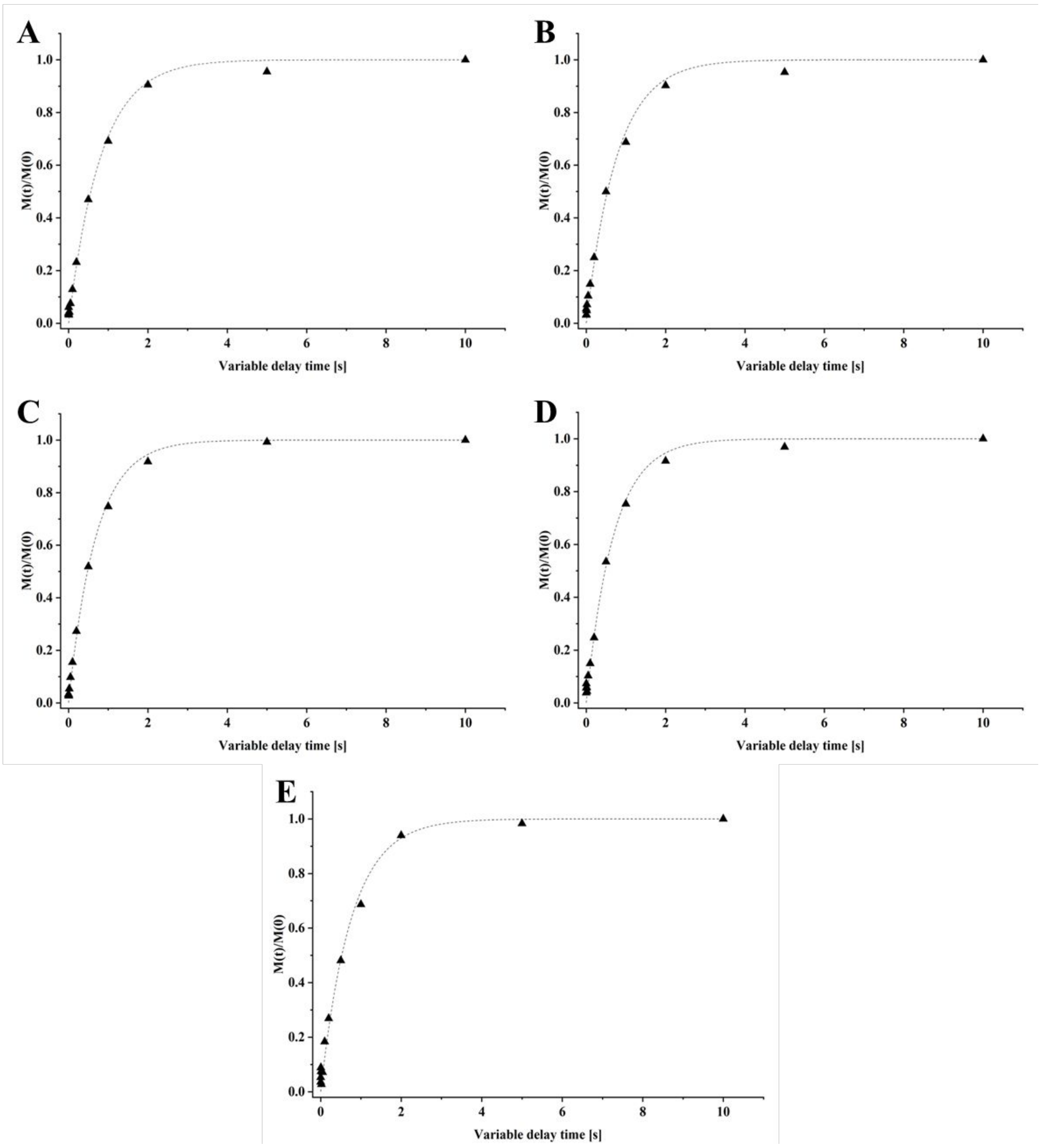

Figure.S4-8 The plot obtained from peak intensity versus variable delay time and fitting curve simulated from $M_{(t)}/M_{(0)} = 1 - e^{-\frac{t}{T_1^{\mathrm{H}}}}$ of WPU.

A) WPU -CH_2- (25.3 ppm), B) WPU -CH_2- (28.4 ppm), C) WPU -CH_2- (31.6 ppm),

D) WPU -CH_2- (33.7 ppm), E) WPU -CH_2-O- (64.9 ppm).
